# Genome-wide expression profiling of microRNAs in poplar upon infection with the foliar rust fungus *Melampsora larici-populina*

**DOI:** 10.1186/s12864-015-1891-8

**Published:** 2015-09-15

**Authors:** Min Chen, Zhimin Cao

**Affiliations:** College of Forestry, Northwest A & F University, Yangling, Shaanxi 712100 People’s Republic of China

**Keywords:** *Populus szechuanica*, MicroRNA, High-throughput sequencing, *Melampsora larici-populina*

## Abstract

**Background:**

MicroRNAs (miRNAs) are small non-coding RNAs that regulate the gene expression of target mRNAs involved in plant growth, development, and abiotic stress and pathogen responses. Previous studies have reported miRNAs in *Populus* that respond to abiotic stresses, such as cold, heat, drought, flooding, high salt and mechanical stress. However, little is known about the regulatory roles of these molecules in the *Populus* response to the stress of foliar rust fungal infection. Here, we identified the miRNA profiles of *Populus* after inoculation with *Melampsora larici-populina* using high-throughput sequencing and bioinformatics analysis. Quantitative real-time PCR (qRT-PCR) was used to validate the expression levels of 10 miRNAs.

**Results:**

A total of 90 known miRNAs belonging to 42 families and 378 novel miRNAs were identified from three small-RNA libraries of *Populus szechuanica* infected with *M. larici-populina* isolates Sb052 and Th053 and a control. Comparative analysis revealed that the expression of 38 known miRNAs and 92 novel miRNAs in *P. szechuanica* after infection with different rust fungus isolates showed significant differences, and more miRNAs were suppressed during rust infection. Among the differentially expressed miRNAs, 7 known and 20 novel miRNAs were relevant to the rust fungus infection, and according to KEGG (Kyoto Encyclopaedia of Genes and Genomes) pathway analysis, these miRNAs primarily regulate genes encoding disease-resistance proteins, serine/threonine protein kinases, transcription factors, and related proteins. QRT-PCR analysis indicated that most miRNAs were up-regulated in the Sb052 library and down-regulated in the Th053 library at 48 h post-inoculation (hpi).

**Conclusions:**

These results demonstrate that the expression of miRNAs was altered in poplar under stress associated with *M. larici-populina* infection, and different temporal dynamics were observed in incompatible and compatible libraries. These findings suggest important roles for miRNA regulation in *Populus* upon infection with foliar rust fungus.

**Electronic supplementary material:**

The online version of this article (doi:10.1186/s12864-015-1891-8) contains supplementary material, which is available to authorized users.

## Background

MiRNAs, small endogenous non-coding RNAs approximately 21–24 nucleotides (nt) in length, play an important role in regulating gene expression at the post-transcriptional level [1]. A large number of miRNAs have recently been identified in plants, and numerous miRNAs have been entered into the miRBase. miRNAs are involved in regulating growth, development, hormone balance, floral morphogenesis, reproductive performance, and biotic and abiotic stress responses. Upon nutritional deficiency, miRNAs regulate the metabolic balance of phosphorus, sulphur and copper in plants [2–5]. Under environmental stresses, including drought, flooding, salt, cold and heat, stress-regulated miRNAs in plants confer resistance to the extreme conditions [6]. In addition, the expression of miRNAs can alter in plants in response to biotic stresses, such as fungi, bacteria, viruses, or insects [7–16].

The genome of *P. trichocarpa* is small, and these plants reach reproductive maturity in a relatively short time [17, 18]. Thus, *Populus* is a useful forest species model for genetic and ecological research. In recent years, studies on *Populus* miRNAs have increased [19–21], particularly the expression of miRNAs and the function of the targets in response to abiotic stresses, including cold, heat, drought, flooding, high salt and mechanical stress [22–29]. A larger number of serious diseases occur in poplar, particularly foliar rust disease induced through infections with the rust fungus *Melampsora* spp., which markedly affects the growth of seeding and saplings. However, few studies have focused on miRNA expression profiling in the response of poplars to biotic stress, particularly pathogen stress [30, 31].

To understand the roles of miRNAs in the response of poplars to pathogenic fungi, we constructed three libraries from uninfected *P. szechuanica* leaves (control) and leaves infected with avirulent and virulent isolates of *M. larici-populina*, detected the expression of miRNAs through high-throughput sequencing and analysed the target genes. QRT-PCR was used to analyse the expression of ten miRNAs in plants inoculated for different times. The results of the present study help elucidate the regulatory mechanisms of *Populus* in response to foliar rust fungal infections.

## Results

### Analysis of miRNA sequences

Using Solexa sequencing, we constructed three libraries from uninoculated *P. szechuanica* leaves (control) and leaves inoculated with avirulent (Sb052) and virulent (Th053) isolates of *M. larici-populina*. A total of 17,801,357, 19,602,945 and 17,962,927 raw reads were obtained from Sb052, Th053 and control libraries, respectively (Table 1). After eliminating low-quality reads and impurities, 17,754,456, 19,544,426 and 17,918,326 high-quality reads were obtained from the three libraries. After discarding 3’ adapter deletions, insertion deletions, 5’ adapter contaminants, poly-A sequences and sequences less than 18 nt from the high-quality reads, 17,557,485, 19,398,402 and 17,764,475 clean reads were used for further analysis. The proportions of clean reads were 98.89, 99.25 and 99.14 % of the total reads obtained from the three libraries, respectively.

**Table 1:** Summary of small-RNA sequencing data in three *P. szechuanica* libraries

Type	Sb052		Th053		Control	
	Count	Percent	Count	Percent	Count	Percent
total_reads	17801357		19602945		17962927	
high quality	17754456	100 %	19544426	100 %	17918326	100 %
3’adapter_null	3591	0.02 %	5632	0.03 %	2971	0.02 %
Insert_ null	3869	0.02 %	4910	0.03 %	2943	0.02 %
5’adapter_contaminants	75547	0.43 %	98975	0.51 %	98892	0.55 %
smaller_than_18nt	112591	0.63 %	35223	0.18 %	47711	0.27 %
Poly-A	1373	0.01 %	1284	0.01 %	1334	0.01 %
clean reads	17557485	98.89 %	19398402	99.25 %	17764475	99.14 %

**Fig. 1:**
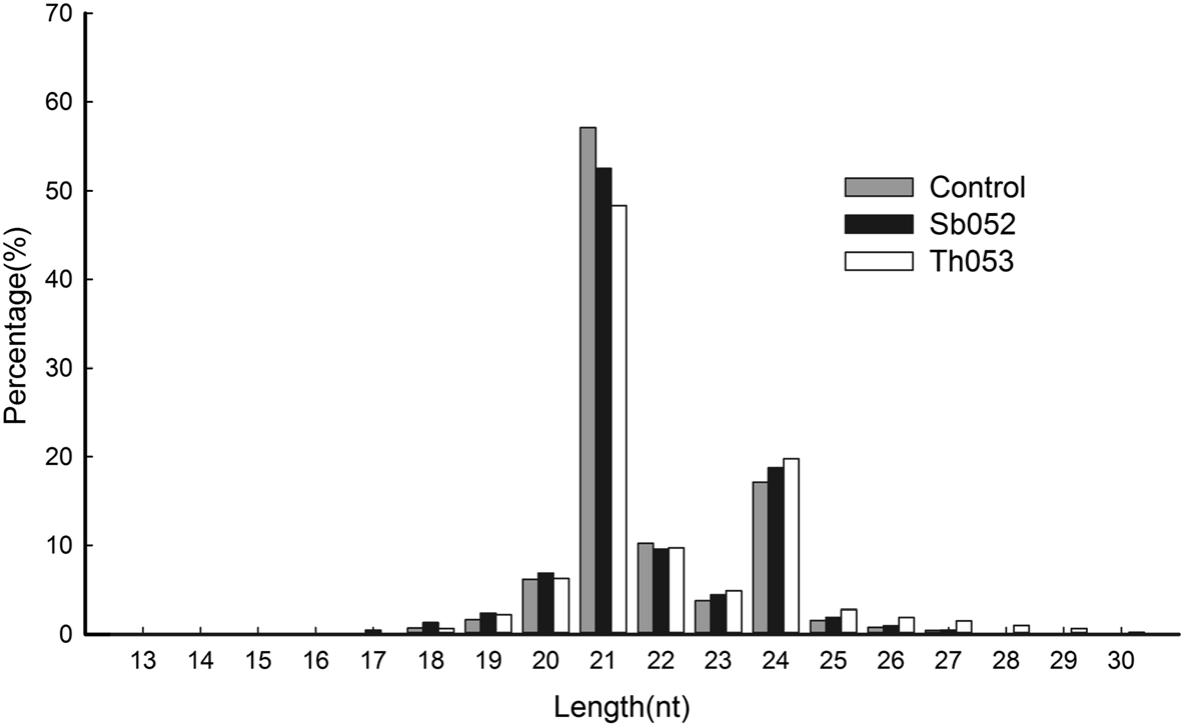
Length, distribution and frequency of sRNAs in the three *P. szechuanica* libraries

The small RNA (sRNA) reads were typically 19 to 25 nt in length (Fig. 1). Among these sequences, 21 nt sRNAs were the most abundant in the three libraries, accounting for 57.15 (Control), 52.55 (Sb052) and 48.34 % (Th053) of the total reads, followed by 24 nt sRNAs, which accounted for 17.13, 18.76 and 19.77 % of the total reads, respectively. These results were consistent with previous studies in *Populus* [24, 25, 27, 28]. However, it was also reported that the most abundant reads length was 24 nt, followed by 21 nt [31, 32], indicating that the miRNAs in *Populus* are primarily 21 and 24 nt in length. Furthermore, we observed that the number of 21 nt sequences in the control libraries was more abundant than that in the treatment libraries, and the number of 24 nt sequences in the treatment libraries was more abundant than that in the control libraries. The results suggest that the number of 21 nt miRNA sequences decreased and the number of 24 nt miRNA sequences increased in *P. szechuanica* after infection with foliar rust fungus.

More than ten million total sRNA sequences and nearly three million unique sRNA sequences were identified (Table 2). Obviously, the sRNA sequences in the three libraries were sufficiently abundant to provide adequate data for further analysis. Unannotated sequences accounted for 30.99, 30.56 and 32.33 % of the total clean reads and 81.78, 80.95 and 81.21 % of the unique reads in Sb052, Th053 and control libraries, respectively. Thus, unknown sequences accounted for substantial proportions of the RNA categories in each library.

**Table 2:** Distribution of sRNAs in different categories in three libraries of *P. szechuanica*

Category	Sb052		Th053		Control	
	Unique sRNAs (%)	Total sRNAs (%)	Unique sRNAs (%)	Total sRNAs (%)	Unique sRNAs (%)	Total sRNAs (%)
Total	2905624(100)	17557485(100)	3287847(100)	19398402(100)	2797933(100)	17764475(100)
exon	136541 (4.70)	576314(3.28)	139463(4.24)	553903(2.86)	136964(4.89)	674443(3.80)
intron	109233(3.76)	455077(2.59)	111882(3.40)	441890(2.31)	109735(3.93)	472656(2.66)
miRNA	35186(1.21)	7360137(41.92)	38672(1.18)	7664446(39.51)	36008(1.29)	7911943(44.54)
rRNAetc^a^	247466(8.52)	3721683(21.2)	335307(10.21)	4799682(24.75)	242107(8.65)	2958184(16.65)
repeat	854(0.03)	3168(0.02)	968(0.03)	3076(0.02)	874(0.03)	3268(0.02)
unann^b^	2376344(81.78)	5441106(30.99)	2661555(80.95)	5929105(30.56)	2272245(81.21)	5743981(32.33)

^a^rRNAetc included rRNA, snRNA, snoRNA and tRNA

^b^unannotated reads

### Known miRNAs in *P. szechuanica*

**Fig. 2:**
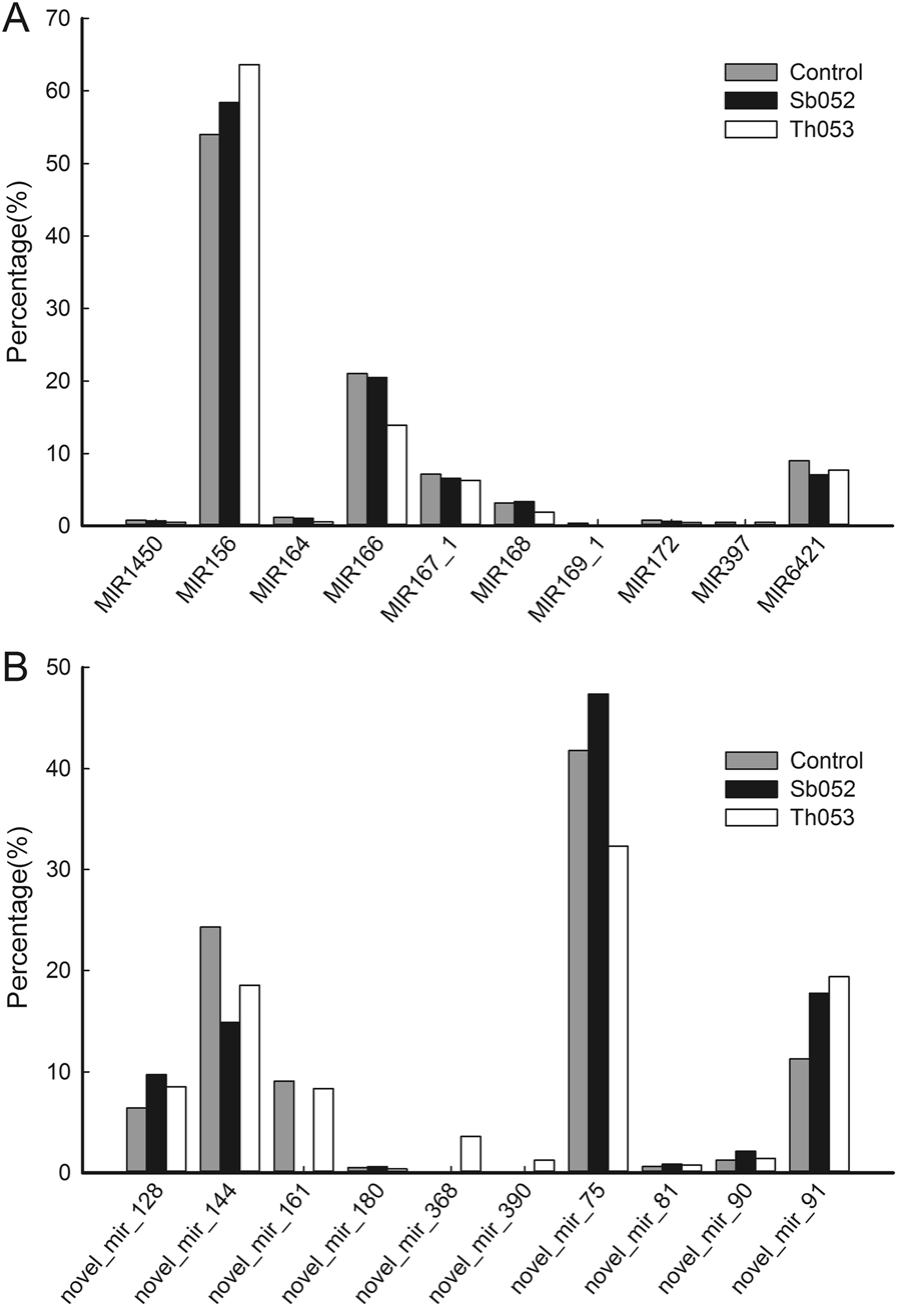
Abundance of highly expressed miRNAs in the three *P. szechuanica* libraries*.* **a** The percentages of 10 known miRNA families accounting for the total known reads. **b** The percentages of 10 novel miRNAs accounting for the total novel reads

Known miRNAs in *P. szechuanica* infected with *M. larici-populina* were identified through homologous alignment analysis using the plant miRNA in miRBase 20.0. A total of 90 known miRNAs were obtained in the Sb052, Th053 and control libraries, belonging to 42 families from 313 genomic loci (Additional file 1: Table S1). The percentages of dominant miRNA families are shown in Fig. 2a, and most of these miRNAs were largely conserved in various plant species, except miR6421, which was only identified in *P. trichocarpa.* The expression levels of a few miRNA families, such as MIR156 and MIR166, were obviously high in three libraries. The most abundant miRNA family was MIR156, accounting for 58.37, 63.61 and 54.02 % of the total sequences in Sb052, Th053 and control libraries, respectively.

miRNAs have a broad range of expression, varying from several to millions of reads. For example, miR157 generated 3,196,614 reads, whereas miR2275 generated only 11 reads in the control library. Most of the conserved miRNAs were identified from the three libraries, and certain miRNAs were abundant in some samples but scarce or even lacking in other samples. For example, the expression of miR4414 in Sb052 and Th053 libraries generated 82 and 118 reads, respectively; however, this miRNA was not present in the control library. Similarly, although the expression of miR2118 generated 7532 reads in the control library, this miRNA generated no reads in the other two libraries. Moreover, the number of reads for different members of same family varied widely. For example, miR473 and miR477 both belong to the miR477 family; however, 17,922 reads were obtained for miR473, whereas only 633 reads were obtained for miR477 in the control library.

**Fig. 3:**
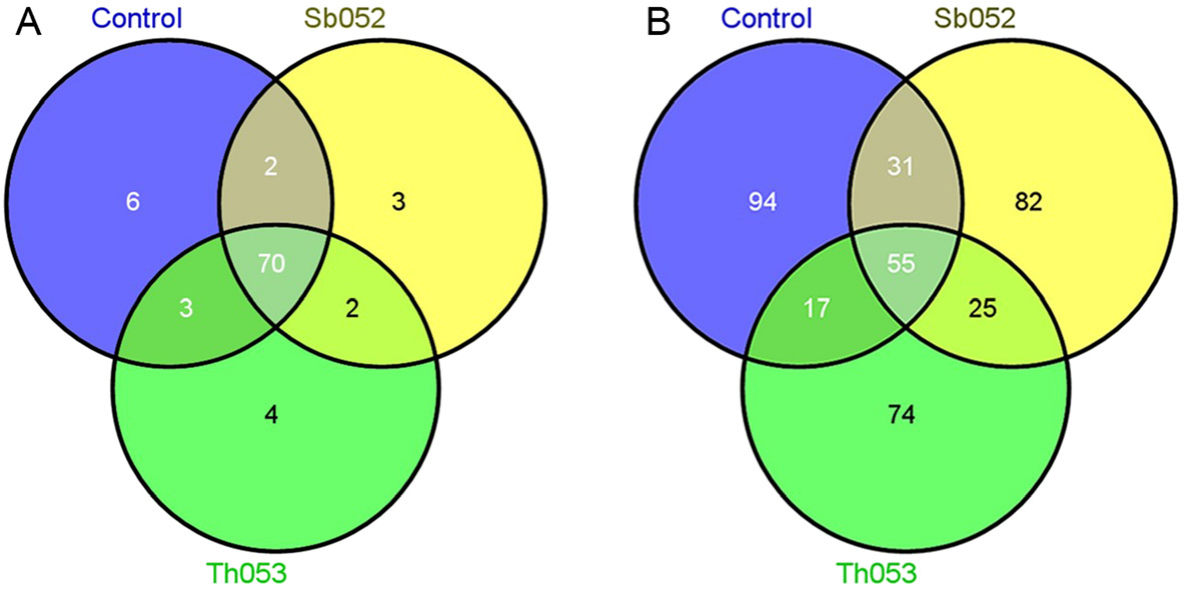
Venn diagrams of the miRNAs identified in the three libraries. **a** Known miRNAs. **b** Novel miRNAs

A total of 70 miRNAs were common to the three libraries examined. Three miRNAs (miR5172, miR6446 and miR6465) were only identified in the Sb052 library, whereas four miRNAs (miR1863, miR476, miR6300 and miR7825) were specific to the Th053 library (Fig. 3a).

### Novel miRNAs in *P. szechuanica*

The unique difference between miRNA and other small-molecule RNAs is that miRNA precursors form stem-loop structures as the basis for the prediction of new miRNA. In the present study, 378 novel miRNAs from 508 genome loci were identified using Mireap software (Beijing Genomics Institute (BGI), Shenzhen, China) (Additional file 2: Table S2). The precursor sequences and predicted secondary structures of 20 novel miRNAs are shown in Additional file 3: Figure S1. A total of 56 miRNAs were generated from multiple loci, among which novel_mir_16 had 13 loci. Marked differences in the abundance of the novel miRNAs were also observed. The percentages of highly expressed novel miRNAs are shown in Fig. 2b. The most abundant miRNA was novel_mir_75, with 59,661, 35,565 and 81,373 reads corresponding to 47.36, 32.32 and 41.77 % of the total reads in the Sb052, Th053 and control libraries, respectively, followed by novel_mir_144 and novel_mir_91. As shown in Fig. 3b, 55 novel miRNAs were common to the three libraries, whereas 82 miRNAs were unique to the Sb052 library and 74 miRNAs were only identified in the Th053 library.

### Differential expression of miRNAs within inoculation treatments

The analysis of the differential expression of miRNAs in the inoculated and control libraries showed that 32 miRNAs (5 known and 27 novel miRNAs) were up-regulated and 40 miRNAs (15 known and 25 novel miRNAs) were down-regulated in *P. szechuanica* infected with Sb052, whereas 35 miRNAs (12 known and 23 novel miRNAs) were up-regulated and 53 miRNAs (15 known and 38 novel miRNAs) were down-regulated in *P. szechuanica* infected with Th053. The comparison of the miRNA expression levels in *P. szechuanica* inoculated with Sb052 and Th053 showed that 24 known (6 up-regulated and 18 down-regulated) and 52 novel miRNAs (33 up-regulated and 19 down-regulated) were differentially expressed between the two libraries. In brief, we identified 38 known and 92 novel differentially expressed miRNAs from the three libraries that might play important roles in response to pathogen stress (Additional file 4: Table S3). Among these miRNAs, miR2118, miR482, novel_mir_166 and novel_mir_198 were suppressed or down-regulated, while miR472, novel_mir_250 were up-regulated in both Sb052 and Th053 libraries compared to the control library. The expression level of miR1444, miR397 and novel_mir_191 were down-regulated in the Sb052 library, but their sequencing reads did not vary significantly in the Th053 library. In contrast, expression levels of novel_mir_248, novel_mir_290 and novel_mir_357 were up-regulated in the Sb052 library, but these miRNAs were not expressed in either the Th053 or the control library. The expression levels of miR164, novel_mir_11, novel_mir_211 and novel_mir_244 did not vary significantly in the Sb052 library, however, in the Th053 library, they were suppressed. In conclusion, the expressions levels of the same miRNAs were significantly different, and the expression levels of poplar miRNAs were more suppressed during rust infection.

### Target prediction and functional analysis

**Fig. 4:**
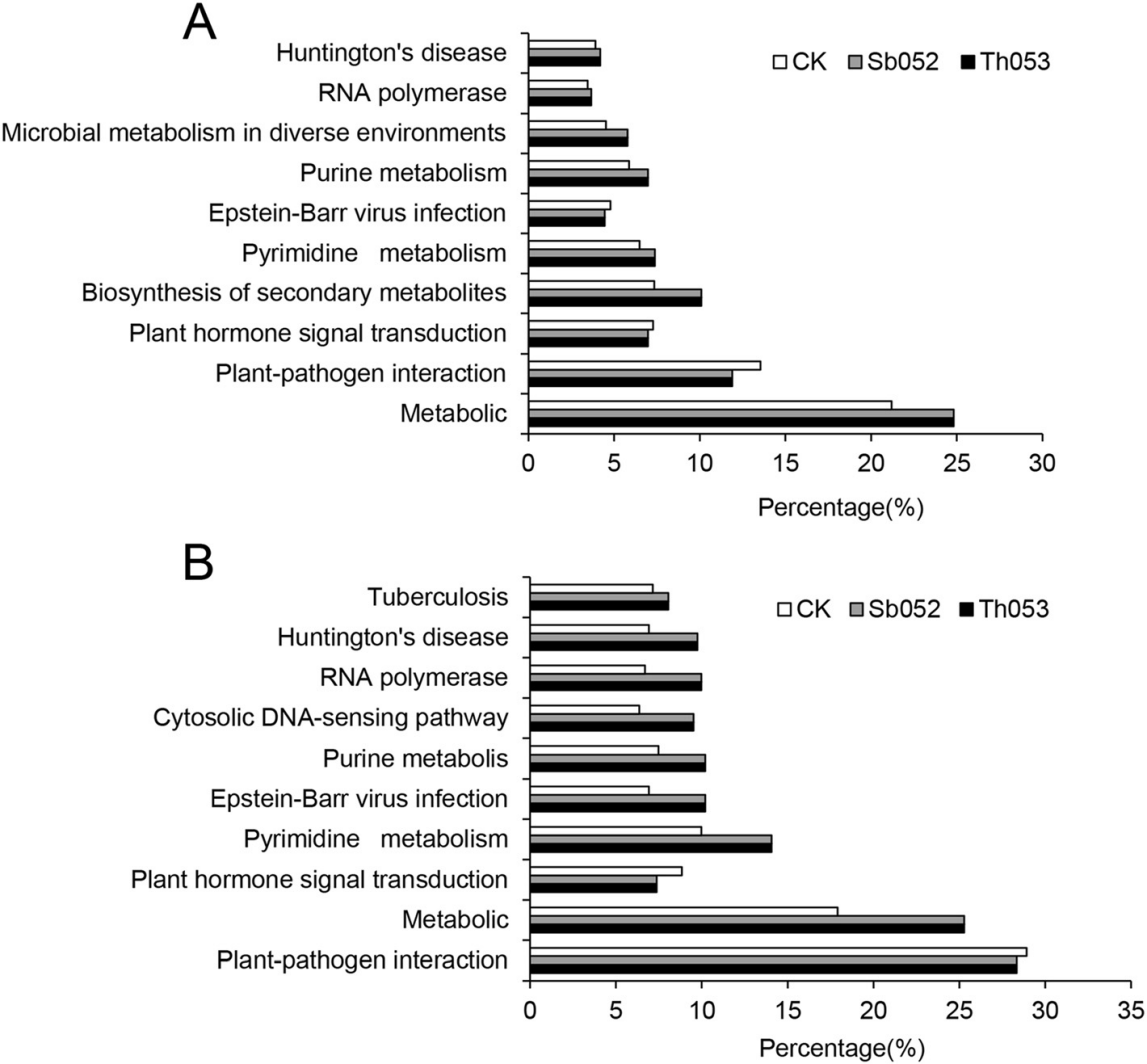
Top ten preferential pathways from KEGG pathway analysis of the target genes in the three *P. szechuanica* libraries. **a** Known miRNAs. **b** Novel miRNAs. X-axis represents the percentages of target genes involved in the ten pathways accounting for the total targets

The majority of plant miRNAs were completely complementary with their targets, which provided a convenient starting point for the prediction of target genes. A large number of targets were predicted for most miRNAs using the program developed by BGI (Shenzhen, China) and obeying the criteria and methods outlined previously [33, 34]. The results of KEGG pathway analysis revealed that target genes of these miRNAs were involved in various biological and biochemical processes in plant growth and development. The top ten preferential pathways of the target genes in the three *P. szechuanica* libraries were shown in Fig. 4. As for the known miRNAs, the pathway that accounted for the largest percentage of the total targets was metabolic pathways, followed by plant-pathogen interaction (Fig. 4a). However, regarding novel miRNAs, the pathway with the highest proportion of target genes was plant-pathogen interaction. Metabolic pathways took second place (Fig. 4b). Differentially expressed miRNAs and their target gene functional annotations were list in Additional file 4: Table S3. Among these miRNAs, twenty-seven miRNAs including 7 known miRNAs and 20 novel miRNAs in the leaves of *P. szechuanica* infected with *M. larici-populina*, were analysed and the results showed that the target genes were primarily associated with disease-resistance proteins, serine/threonine protein kinases, transcription factors, and related proteins (Table 3). The genes of disease resistance proteins RPS2, RPS5 and RPM1 were putative targeted by 3 known miRNAs (miR2118, miR472, miR482) and 8 novel miRNAs (novel_mir_11, novel_mir_166, novel_mir_211, novel_mir_244, novel_mir_248, novel_mir_250, novel_mir_357 and novel_mir_403). LRR receptor-like serine/threonine-protein kinase (FLS2) was predicted to be only targeted by several novel miRNAs, novel_mir_191, novel_mir_198, novel_mir_368, novel_mir_93. Two transcription factors involving in plant defense responses, transcription factor WRKY and MYC2, were found to be targeted by novel_mir_290 and miR530, respectively. In addition, polyphenol oxidase, L-ascorbate oxidase and peroxidase genes were also predicted to be targeted by miR1444, miR397 and novel_mir_191, respectively. Meanwhile, we also observed that some miRNAs have identical and unique targets; for example, the targets of the miR472, miR482, miR2118, novel_mir_11 and novel_mir_357 were all disease-resistance proteins, whereas some miRNAs, such as miR530, novel_mir_191, novel_mir_248, novel_mir_250 and novel_mir_270 have multiple target genes and functional annotations, including the genes of various enzymes, disease-resistance proteins and transcription factors.

**Table 3:** Disease resistance-related miRNAs from *P. szechuanica* and target gene functional annotations

miRNA name	Target accession	Functional annotation
miR1444	Potri.011G047300.1,Potri.011G108200.1,Potri.011G108300.1,Potri.001G387900.1,Potri.001G388100.1,Potri.001G388300.1,Potri.001G388400.1,Potri.001G388800.1,Potri.001G388800.2,Potri.001G388900.1,Potri.T061900.1,Potri.T062100.1,Potri.T062200.1	polyphenol oxidase
miR2118	Potri.T003600.1,Potri.T003900.1,Potri.T004100.2,Potri.T045200.1,Potri.T004200.1,Potri.018G136300.1,Potri.T014600.1,Potri.T012900.1	disease resistance protein RPS2
		disease resistance protein RPS5
	Potri.T004100.1,Potri.005G041100.2,Potri.005G041300.1,Potri.005G041100.1,Potri.005G042400.2,Potri.005G042400.1,Potri.005G042900.1,Potri.T016200.1,Potri.T016200.2	disease resistance protein RPS2
	Potri.T068700.1,Potri.011G011900.1,Potri.011G013800.1	disease resistance protein RPM1
	Potri.011G013100.1,Potri.011G015300.1	disease resistance protein RPM1,
		disease resistance protein RPS2
	Potri.008G220200.1,Potri.008G220200.2	disease resistance protein RPM1
		disease resistance protein RPS5
miR394	Potri.018G095700.1,Potri.018G095700.2	glycerol kinase
miR397	Potri.010G183600.1,Potri.010G183600.2,Potri.009G034500.1,Potri.009G042500.1,Potri.009G102700.1,Potri.009G156600.1,Potri.009G156800.1,Potri.004G156400.2,Potri.004G156400.1,Potri.011G120200.1,Potri.011G120300.1,Potri.006G087100.1,Potri.006G094100.1,Potri.006G096900.1,Potri.006G097000.1,Potri.006G097100.1,Potri.016G106000.1,Potri.016G107500.1,Potri.016G112000.2,Potri.016G112000.1,Potri.016G112100.1,Potri.001G054600.1,Potri.001G184300.1,Potri.001G248700.1,Potri.001G401100.1,Potri.001G401300.1	L-ascorbate oxidase
miR472	Potri.T003600.1,Potri.T003900.1,Potri.T004100.2,Potri.T004200.1,Potri.T045200.1,Potri.T012900.1,Potri.018G136300.1,Potri.T014600.1	disease resistance protein RPS2
		disease resistance protein RPS5
	Potri.T068700.1,Potri.011G013800.1	disease resistance protein RPM1
	Potri.011G013100.1,Potri.011G015300.1	disease resistance protein RPM1
		disease resistance protein RPS2
	Potri.T016200.1,Potri.T016200.2,Potri.T004100.1	disease resistance protein RPS2
miR482	Potri.017G015300.2,Potri.017G015300.1,Potri.017G015600.2,Potri.017G015600.4,Potri.017G015600.3,Potri.017G015600.1,Potri.004G196100.1,Potri.003G199100.1,Potri.003G199600.1,Potri.003G200800.1	disease resistance protein RPM1
	Potri.001G427700.2,Potri.001G427700.1,Potri.001G432200.1	disease resistance protein RPS2
	Potri.001G432200.1	disease resistance protein RPS5
miR530	Potri.014G099700.1	transcription factor MYC2
novel_mir_11	Potri.004G169800.1,Potri.004G169800.2,Potri.004G170300.1,Potri.003G017200.1,Potri.003G026900.1,Potri.018G136100.1,Potri.014G001900.1,Potri.014G002000.1,Potri.014G002200.1,Potri.014G002300.1,Potri.014G003200.2,Potri.014G003200.1,Potri.014G003400.1,Potri.014G003600.2,Potri.014G003600.1,Potri.014G005600.2,Potri.014G005600.1,Potri.014G007600.1,Potri.014G009300.1,Potri.014G009400.1,Potri.014G009400.2,Potri.014G009600.1,Potri.014G010600.1,Potri.014G010700.1,Potri.014G010900.1,Potri.014G012000.1	disease resistance protein RPM1
novel_mir_118	Potri.006G047300.1,Potri.006G047300.2	calcium-binding protein CML
novel_mir_166	Potri.013G098000.1,Potri.013G097900.1	disease resistance protein RPM1
novel_mir_191	Potri.018G035100.1	LRR receptor-like serine/threonine-protein kinase FLS2
	Potri.019G103900.1,Potri.019G103900.2,Potri.019G103900.3	serine/threonine-protein kinase PBS1
	Potri.009G115200.1,Potri.009G115200.2	brassinosteroid insensitive 1-associated receptor kinase 1
novel_mir_198	Potri.005G031900.1	LRR receptor-like serine/threonine-protein kinase FLS2
novel_mir_244	Potri.017G103500.1,Potri.017G103500.2, Potri.014G063500.1	disease resistance protein RPM1
	Potri.014G064300.1	disease resistance protein RPM1
		disease resistance protein RPS2
		disease resistance protein RPS5
novel_mir_248	Potri.T049000.1	disease resistance protein RPM1
		disease resistance protein RPS2
novel_mir_206	Potri.012G054700.1,Potri.012G054700.2	chitin elicitor receptor kinase 1
novel_mir_211	Potri.017G127200.1,Potri.017G127800.1,Potri.017G128000.2,Potri.017G128000.1,Potri.017G133600.1,Potri.017G133700.1,Potri.017G136400.1,Potri.017G136700.1,Potri.017G136900.1,Potri.017G138100.1,Potri.018G080500.1,Potri.018G080700.1,Potri.T105500.1,Potri.001G033100.1,Potri.T096100.1	disease resistance protein RPM1
novel_mir_222	Potri.013G108200.2 Potri.013G108200.1	cyclic nucleotide gated channel, other eukaryote
novel_mir_25	Potri.012G054700.1,Potri.012G054700.2	chitin elicitor receptor kinase 1
novel_mir_250	Potri.019G018800.1,Potri.T068600.1,Potri.011G008600.1,Potri.011G011600.1,Potri.011G060600.1,Potri.011G008700.1,Potri.011G013400.1,Potri.011G015400.1,Potri.006G269900.1	disease resistance protein RPM1
	Potri.T037600.1,Potri.T039900.1,Potri.T039100.1,Potri.T039300.1,Potri.006G270000.1	disease resistance protein RPS2
		disease resistance protein RPM1
	Potri.T067500.1	disease resistance protein RPM1
		disease resistance protein RPS5
	Potri.T112200.1,Potri.001G363400.1	disease resistance protein RPM1
		disease resistance protein RPS2
novel_mir_270	Potri.005G231100.2	extracellular signal-regulated kinase 1/2
novel_mir_290	Potri.001G361600.1,Potri.001G361600.2, Potri.001G361600.3	WRKY transcription factor 33
novel_mir_357	Potri.T003600.1,Potri.T044800.1,Potri.T045300.2,Potri.T045300.2,Potri.T045300.1,Potri.T045300.1,Potri.T024700.1,Potri.T024900.1,Potri.T025100.1,Potri.T025800.1,Potri.T025800.1,Potri.T026400.1,Potri.T026800.1,Potri.T026700.1,Potri.T026900.1,Potri.T027200.1,Potri.T027500.1,Potri.T027300.1,Potri.T027700.1,Potri.T028100.1,Potri.T028300.1,Potri.019G022800.1,Potri.019G020500.1,Potri.019G020200.2,Potri.019G020200.1,Potri.019G014500.1,Potri.018G137900.1,Potri.018G136500.1,Potri.018G135600.1,Potri.018G136700.1,Potri.T015900.2,Potri.T015900.1,Potri.T013900.1,Potri.T013800.1,Potri.T013300.3,Potri.T013300.2,Potri.T053000.1,Potri.001G406000.1,Potri.T012000.1,Potri.T013300.1,Potri.T014600.1	disease resistance protein RPS2
		disease resistance protein RPS5
	Potri.T045000.1,Potri.T046000.1,Potri.T025300.1,Potri.T026200.1,Potri.T026600.1,Potri.011G127300.2,Potri.T028700.1,Potri.T028000.1,Potri.011G127300.1,Potri.T014900.1,Potri.019G002800.1,Potri.019G002800.2	disease resistance protein RPS2
novel_mir_368	Potri.011G104700.1,Potri.011G104900.1,Potri.T085700.2,Potri.T085700.3,Potri.T085700.1,Potri.T086100.3,Potri.T086100.1,Potri.T086100.2	LRR receptor-like serine/threonine-protein kinase FLS2
novel_mir_38	Potri.007G128600.1	calcium-binding protein CML
novel_mir_387	Potri.004G025200.1,Potri.004G026100.1	serine/threonine-protein kinase PBS1
novel_mir_403	Potri.T001600.1	disease resistance protein RPM1
novel_mir_93	Potri.003G041600.1	LRR receptor-like serine/threonine-protein kinase FLS2

### QRT-PCR validation of *P. szechuanica* miRNAs

**Fig. 5:**
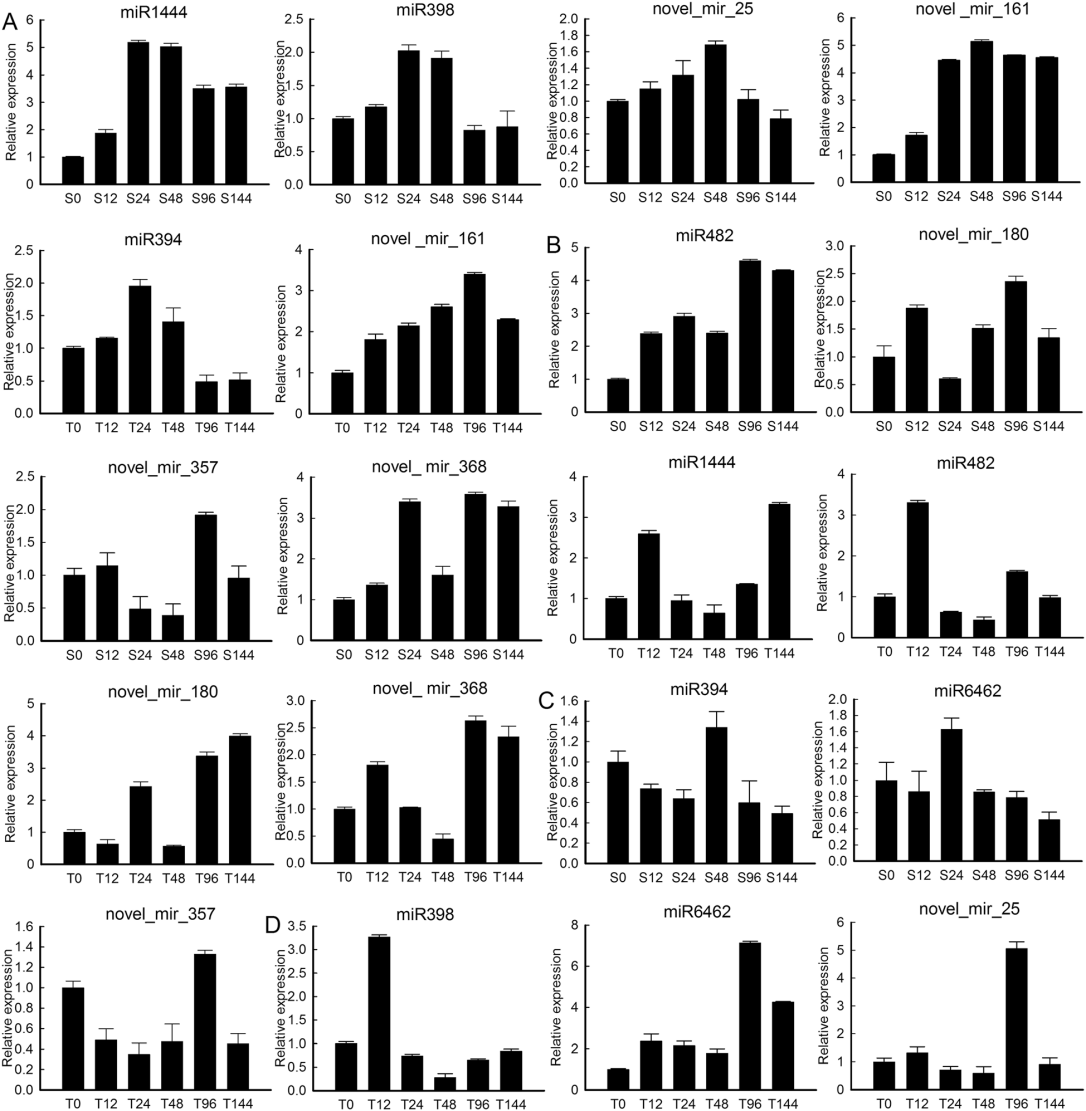
Temporal dynamic expression of miRNAs in *P. szechuanica* infected with *M. larici-populina* at 0, 12, 24, 48, 96 and 144 hpi. When plants were infected with Sb052(S), the validated miRNAs were classified into expression patterns **a**, **b** and **c**. When plants were infected with Th053 (T), the validated miRNAs were classified into expression patterns **a**, **b**, **c** and **d**. For normalization, 5.8S rRNA was selected as a reference gene, and the expression level at 0 hpi (S0 and T0) was set to 1. The statistical analyses and graphics were generated using the SigmaPlot 12.5 statistical package

To confirm the temporal dynamic expression of miRNAs involved in pathogen stress, we further analysed 10 miRNAs in *P. szechuanica* infected with *M. larici-populina* at 0, 12, 24, 48, 96, and 144 hpi using qRT-PCR. The results showed that the miRNA expression levels dramatically changed during the pathogenesis of foliar rust infection (Fig. 5). When plants were infected with Sb052, the miRNA expression levels exhibited three patterns: Pattern A, including miR1444, miR398, novel_mir_25, and novel_mir161, gradually increased at the early stage, peaked at 24 or 48 hpi and then gradually declined; Pattern B, including miR482, novel_mir_180, novel_mir_357, and novel_mir_368, initially increased, then decreased, and subsequently increased again, peaking at 96 hpi; Pattern C, including miR394 and miR6462, initially decreased, then increased, peaking at 48 hpi, and subsequently declined again. When plants were infected with Th053, the miRNA expression levels were classified into four patterns. The first three patterns were identical to those observed with Sb052: Pattern A included miR394 and novel_mir_161; Pattern B included miR1444, miR482, novel_mir_180, and novel_mir_368, whose expression markedly declined, reaching the lowest point at 48 hpi; and Pattern C only included novel_mir_357. Pattern D, including miR398, miR6462 and novel_mir_25, suddenly increased, peaking at 12 or 96 hpi, followed by a dramatic decline. In the present study, the sequencing results showed that novel_mir_368 and novel_mir_161 were only observed in the Th053 library or in both the Th053 library and control, respectively; however, these miRNAs could be detected in the Sb052 library using qRT-PCR analysis. These different results suggest that deep sequencing was not sufficient for the detection of the expression of all miRNAs. Interestingly, we observed that the expression of 10 miRNAs, except miR394, novel_mir_161, and novel_mir_357, increased at 48 hpi in the Sb052 library and decreased at 48 hpi in the Th053 library compared with expression levels at 0 hpi, indicating that the expression levels of the same miRNAs differed in incompatible and compatible interaction libraries.

## Discussion

### Identification of miRNAs in *P. szechuanica*

Several studies concerning miRNA expression in *Populus* subjected to various stresses have recently been conducted, and the number of miRNAs identified in *Populus* is increasing. A total of 405 mature *Populus* miRNA sequences have been registered in miRBase 20.0, among which 401 miRNAs have been identified in *P. trichocarpa* and 4 miRNAs have been identified in *P. euphratica.* However, there are few studies concerning the expression of miRNAs under pathogen stress in *Populus*. In the present study, we used high-throughput sequencing to construct libraries from the uninfected leaves of *P. szechuanica* and leaves infected with *M. larici-populina* avirulent isolate Sb052 and virulent isolate Th053. Sequencing obtained 90 known miRNAs belong to 42 families (Additional file 1: Table S1) and 378 novel miRNAs (Additional file 2: Table S2). The known miRNAs were matched with miRBase 20.0, and 43 miRNAs were identified in *Populus*, of which 41 miRNAs were *P. trichocarpa* miRNAs and 2 miRNAs were specific to *P. euphratica*. The remaining miRNAs were conserved in other plant species. Among the conserved miRNAs, MiR156 and MiR166 families were the most abundant, similar to their expression levels in *P. euphratica* [26], *P. tomentosa* [28] and *P. beijingensis* [31], whereas novel miRNAs showed low expression levels compared with the known miRNAs in *P. szechuanica*. A majority of known miRNAs were common to the three libraries, but most of novel miRNAs were only identified in a certain libraries (Fig. 3). These results showed that the unique miRNAs in *P. szechuanica* were abundant and their expression levels were altered by *M. larici-populina* infection.

### Expression and targets function of miRNAs under pathogen stress

In the present study, 32 up-regulated and 40 down-regulated miRNAs were identified in *P. szechuanica* infected with Sb052, whereas 35 up-regulated and 53 down-regulated miRNAs were identified in plants infected with Th053. These findings revealed that more miRNAs were suppressed during rust infection. Among these miRNAs, miR1444, miR482 and miR2118 were significantly down-regulated upon rust fungus infection, consistent with the role of these miRNAs as negative regulators of disease-resistance proteins [23, 31, 35]. Whereas previously reported expression levels of miR167 [36], miR169 [31], miR172 [30], miR398 [31] were contrary to the results obtained in the present study. This differential expression might reflect different miRNA functions in different poplar-pathogen interactions or different inoculation tissues and durations, suggesting that miRNAs have complex regulatory functions in plants subjected to biotic stresses.

According to the functional analysis of the target genes by KEGG pathway database, the high-expressed known miRNAs were mostly responsible for controlling the basic metabolism pathways, while the low-expressed novel miRNAs were mainly involved in regulating the plant-pathogen interaction (Fig. 4). We can see that plant-pathogen interaction and metabolic pathways were significantly over-represented in poplar upon rust fungal inoculation.

miRNAs and their target genes have multiple functions in different plant species. MiR156 was highly conserved and involved in various stresses. It target genes encoded the nucleotide binding site-leucine rich repeat (NBS-LRR) disease resistance protein [37] and squamosa promoter-binding protein (SPB), known to regulate plant development and growth [22, 30, 38]. However, in our trial, miR156 had 43 targets, among which 6 targets were predicted that encoded ubiquitin C, which is involved in DNA repair, translational control and signal transmission. The differentially expressed miRNAs and the functions of their target genes were shown in Additional file 4: Table S3. The expression of miR168 was up-regulated in *P. beijingensis* [31] and *P. trichocarpa* [30] inoculated with canker disease pathogen as well as *P. szechuanica* inoculated with virulent isolate Th053. Four target genes of miR168 encoded argonaute (AGO) which is closely involved in the biosynthesis and function of miRNA. AGO1 mRNA is involved in a negative feedback regulation through the action of miR168 [31, 39]. Previous reports of the expression of miR167 [36] and miR172 [30] were contrary to the results obtained in the present study. miR167 predicted 7 targets encoding auxin response factor (ARF), which is involved in plant hormone signal transduction pathway [30, 36]. Auxin signal transduction is related to bacterial disease resistance in *Arabidopsis* [40], and fungal disease resistance in *P. trichocarpa* [30]. One target of miR172 encoded APETALA2, which is involved in floral development in many plant species under different stresses [30, 37, 38] and another target gene in loblolly pine infected with fusiform rust encoded an LRR protein kinase [37]. In addition, the target functions of several miRNAs were unknown and the targets of some miRNAs have not been predicted.

To uncover the functions of miRNAs in *P. szechuanica* under rust fungus-induced stress, the miRNA target genes involved in disease resistance were annotated using the KEGG pathway database (Table 3). miR1444 expression was down-regulated in the Sb052 library. The target gene of miR1444 encoded polyphenol oxidase, which is an important enzyme for resistance to biotic and abiotic stresses [23, 32]. The over-expression of polyphenol oxidase in tomato enhanced resistance to the pathogen *Pseudomonas syringae* [41]. Similarly, expressions of miR397 were obviously down-regulated in the incompatible interaction. The target gene L-ascorbate oxidase influenced tobacco growth and defense responses by modulating redox state of the apoplastic ascorbic acid [42]. The expression of miR530 was down-regulated in the Sb052 library but up-regulated in the Th053 library, and the target genes of miR530 encoded the transcription factor MYC2, a member of the plant-pathogen interaction pathway, and 1-phosphatidylinositol-4-phosphate 5-kinase, involved in the defence response of poplars to *M. larici-populina* [43]. The target genes of 11 miRNAs, including 3 known miRNAs and 8 novel miRNAs encoded disease resistance proteins, including RPM1, RPS2 and RPS5. These targets all belong to the largest class of disease resistance proteins, the NBS-LRR class, which confer resistance to bacterial, fungal, or viral pathogens [44–46]. The *Arabidopsis thalian*a RPM1, RPS2 and RPS5 genes confer resistance to the bacterial pathogen *P. syringae* [47–49]. In previous studies, the target genes of miR472 [32, 37], miR482 [31, 37, 50] and miR2118 [33, 51] were also found to encode disease-resistance proteins. These findings imply that these miRNAs are responsive to disease stress in different plants and play similar roles in resistance to pathogen infections.

The results of the qRT-PCR analysis demonstrated that most miRNAs in *P. szechuanica* infected with the avirulent isolate Sb052 and virulent isolate Th053 were up-regulated and down-regulated at 48 hpi, respectively. miR1444 is important in plants under biotic and abiotic stresses [23]. The expression of miR1444 at 24 and 48 hpi was lower than that at other times in compatible interactions; however, miR1444 expression reached high levels at the same times in incompatible interactions. In previous studies, many miRNAs were suppressed during fungus infection in compatible interactions, whereas most miRNAs did not vary with respect to incompatible interactions [38] or the number of down-regulated miRNAs was slightly less than that observed in compatible interactions [35]. Rinaldi et al. [43] elucidated that a great number of transcripts were significantly differentially regulated in incompatible interactions, but almost no transcriptional changes were detected in compatible interactions at 48 hpi. These findings provide us with evidence that 48hpi is a vital period for poplar-rust fungus interactions, and related genes are expressed differentially. According to these results, we speculate that miRNAs could possibly regulate the expression of certain genes and, through the changing expression of miRNAs, could play vital roles in regulating disease-resistance in *Populus* infected with rust. Furthermore, a significant difference exists between compatible and incompatible interactions at 48hpi. Therefore, the regulated functions of miRNAs in *P. szechuanica* infected with virulent and avirulent rust isolates at 48 hpi are complex, and require further investigation to understand the molecular mechanism of poplar-rust interactions.

## Conclusions

In summary, a total of 90 known miRNAs and 378 novel miRNAs were identified from *P. szechuanica* infected with the *M. larici-populina* incompatible isolate Sb052, the *M. larici-populina* compatible isolate Th053 and uninfected controls through high-throughput sequencing. Comparative analysis revealed that the expression of miRNAs was significantly different after infection with different rust isolates, and more miRNAs were suppressed during rust infection. The targets of 27 miRNAs were primarily associated with disease resistance. The qRT-PCR analysis showed that miRNA expression exhibited different temporal dynamics in incompatible and compatible libraries. The regulatory mechanism of poplar miRNAs in response to rust fungus infection should be further studied.

## Methods

### Plant materials and rust fungus inoculation

The urediniospores of *M. larici-populina* isolates were multiplied on 1- to 2-year-old potted *P. purdomii* plants susceptible to all *M. larici-populina* isolates. Based on a previous study, the wild poplar *P. szechuanica* from the Qinling Mountains was used as the experimental host. One-year-old cuttings from *P. szechuanica* branches were potted and grown in the greenhouse for two months, and subsequently, the abaxial surfaces of the leaves were inoculated with a urediniospore suspension (1–2 mg/mL) containing isolates Sb052 (pathotype 1^−^-1) and Th053 (pathotype 3^+^-4) of *M. larici-populina*, forming incompatible and compatible interactions, respectively. In addition, the leaves were treated with running water as a control. The inoculation method has been previously described in Rinaldi et al. [43]. The *P. szechuanica* leaves from plants under the three treatments were separately collected at 0, 12, 24, 48, 96 and 144 hpi, immediately frozen in liquid nitrogen and stored at−80 °C until further analysis.

### Total RNA extraction and sRNA sequencing

Total RNA was extracted from the frozen leaf tissues of inoculated and uninoculated *P. szechuanica* using TRNzol reagent (TIANGEN, Beijing, China) according to the manufacturer’s instructions. Total RNA from each treatment at six time points was pooled with equal quantities to identify as many miRNAs as possible. The pooled RNA was used for sRNA library construction for the three treatments, and sRNA was sequenced using an Illumina-Solexa 1G Genetic Analyzer at the BGI (Shenzhen, China).

### Analysis of sequencing data

Clean reads were obtained from raw reads after removing low-quality and contaminant reads. The clean reads were mapped to the *P. trichocarpa* genome [52] using SOAP software [53]. The non-coding RNAs, including rRNAs, scRNAs, snoRNAs, snRNAs, and tRNAs deposited in the NCBI GenBank database [54] and Rfam (11.0) database [55], were removed. Small RNAs corresponding to the exons and introns of mRNA and repeat sequences were also excluded from further analysis. The remaining sRNA sequences were aligned to the miRBase 20.0 database [56], with a maximum of two mismatches, to identify known miRNAs in *P. szechuanica*. The obtained sequences were used to predict hairpin structures using the BGI (Shenzhen, China) program. The remaining unannotated sRNAs were used to predict novel miRNAs using the prediction software Mireap [57].

### Target prediction of miRNAs and functional analysis of the target genes

Target genes were predicted using the program developed by BGI (Shenzhen, China) and obeying the following rules referring to Allen et al. [33] and Schwab et al. [34]: no more than four mismatches between the sRNA and target gene, no more than two adjacent mismatches in the miRNA/target duplex, no adjacent mismatches at positions 2–12 of the miRNA/target duplex (5’ of miRNA), no mismatches at positions 10–11 of the miRNA/target duplex, no more than 2.5 mismatches at positions 1–12 of the miRNA/target duplex (5’ of miRNA), and the minimum free energy (MFE) of the miRNA/target duplex should be 75 % of the MFE of the miRNA bound to the perfect complement. Target genes were searched using *Populus* transcript information [58]. To better understand the roles of miRNAs in *P. szechuanica* under rust fungus-induced stress, the potential target functions were annotated using the KEGG pathway database [59].

### Differential expression analysis of miRNAs

The miRNA reads in the three libraries were used to analyse differential expression and determine significant differences between the control and treatment libraries. The frequency of miRNAs in the three libraries was normalized to one million to reduce potential errors before calculating the fold-change, *P*-value and ratio. Normalized expression = (actual miRNA counts/total counts of clean reads)*1,000,000. Fold-change = log2 (miRNA normalized read counts in the treatment library/miRNA normalized read counts in the control library.)

The *P*-value was calculated as follows:

$$ P\left(\mathrm{x}\Big|\mathrm{y}\right)=\left(\frac{{\mathrm{N}}_2}{{\mathrm{N}}_1}\right)\frac{\left(\mathrm{x}+\mathrm{y}\right)!}{\mathrm{x}!\mathrm{y}!{\left(1+\frac{{\mathrm{N}}_2}{{\mathrm{N}}_1}\right)}^{\left(\mathrm{x}+\mathrm{y}+1\right)}} $$

$$ C\left(\mathrm{y}\le \mathrm{y}\  \min \Big|\mathrm{x}\right)={\displaystyle \sum_{\mathrm{y}=0}^{\mathrm{y}\le \mathrm{y}\  \min }}\mathrm{p}\left(\mathrm{y}\Big|\mathrm{x}\right) $$

$$ D\left(\mathrm{y}\ge \mathrm{y}\  \max \Big|\mathrm{x}\right)={\displaystyle \sum_{\mathrm{y}\ge \mathrm{y}\  \max}^{\infty }}\mathrm{p}\left(\mathrm{y}\Big|\mathrm{x}\right) $$

In this formula, N_1_ and N_2_ represent the total counts of clean reads in control and treatment library, respectively, and *x* and *y* represent the counts of clean reads of a given miRNA in control and treatment libraries, respectively.

A fold change greater than 1 or less than −1 and a *P*-value less than 0.01 suggested that the difference in the miRNA expression between two libraries was highly significant. When the fold-change was greater 1 or less than−1 and the *P*-value was between 0.01 and 0.05, the expression of the miRNA was significantly different between the two libraries.

The ratio of miRNA normalized read counts in treatment library/miRNA normalized read counts in control library was used to determine changes in the expression of an miRNA in the treatment samples compared with the control sample. When the ratio was more than 2, the miRNA was up-regulated, and when the ratio was less than 1/2, the miRNA was down-regulated.

### Quantitative real-time PCR analysis of miRNA expression

To verify the expression levels of identified miRNAs, qRT-PCR was performed in accordance with Shi and Chiang [60]. Total RNA was extracted from Sb052- and Th053-infected *P. szechuanica* leaves at 0, 12, 24, 48, 96 and 144 hpi, and qRT-PCR was performed using the IQ5 Real-time Quantitative PCR Detection System (Bio-RadLaboratories, California, USA) with the SYBR® PrimeScript™ miRNA RT-PCR Kit (TaKaRa, Dalian, China) according to the manufacturers’ instructions. The reactions were performed in a total volume of 25 μL containing 2.0 μL of diluted cDNA, 1 μL of each primer, and 12.5 μL of SYBR Green premix Ex Taq II with the following reaction conditions: 95 °C for 30 s, followed by 40 cycles of 95 °C for 5 s and 60 °C for 20 s. A melting curve analysis was generated to verify the specificity of the PCR amplification. Each sample was processed in triplicate. Ten miRNAs, including five known and five novel miRNAs, were tested. For normalization, the 5.8S rRNA was used as the reference gene [22] and the expression level at 0 hpi was set to 1. All validated miRNAs and primer sequences are listed in Additional file 5: Table S4.

### Availability of supporting data

The supporting data are included within the article and additional files.

## Additional files

Additional file 1: Table S1. Known miRNAs identified in the three *P. szechuanica* libraries. (XLSX 77 kb) Additional file 2: Table S2. Novel miRNAs identified in the three *P. szechuanica* libraries. (XLSX 110 kb) Additional file 3: Figure S1. Precursor sequences and the predicted secondary structures of the 20 novel miRNAs from *P. szechuanica* (“(”represent base matches, “.” represent base mismatches). (PDF 387 kb) Additional file 4: Table S3. Differentially expressed miRNAs from *P. szechuanica* and target gene functional annotations. (XLSX 31 kb) Additional file 5: Table S4. miRNAs and primer sequences for qRT-PCR. (XLSX 10 kb)

## Abbreviations

miRNAs: microRNAs
qRT-PCR: quantitative real-time PCR
KEGG: Kyoto Encyclopedia of Genes and Genomes
hpi: Hour post-inoculation
nt: Nucleotides
NBS-LRR: Nucleotide binding site-leucine rich repeat
AGO: Argonaute
