# Supplementary material for: Genome-wide expression profiling of microRNAs in poplar upon infection with the foliar rust fungus Melampsora larici-populina
Source: BMC Genomics. 2015 Sep 15;16(1):696. doi: 10.1186/s12864-015-1891-8 (PMC4570220; doi:10.1186/s12864-015-1891-8)

Additional file 3: Figure S1 Precursor sequences and the predicted secondary structures of the 20 novel miRNAs from *P. szechuanica* ("("represent base matches, "." represent base mismatches)

Novel_mir_11 (CK-m0012-5p 19, Sb052-m0006-5p 14, Th053-m0013-5p 9)

CCTCAAGAATTGAAGAGACGTGAAGCCATGAAATTCACCACATGAACATCTCGGTTCATCACA
ATCTTCAATTTCAAATT

......((((((((((...((((((((..((..((((.....))))..))..))))..))))..))))))))))......

*********TGAAGAGACGTGAAGCCATGA**
**

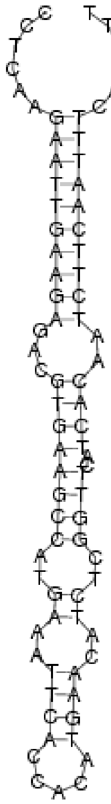

Novel_mir_118 (CK-m0139-3p 15 Sb052-m0134-3p 18)

ATGGACACTAAGAAGTAGAATGTCCTAAACCATAACATAACTGACAATTGGTTTGGTGATTAT
TAGACCACTTATTATTGTAGGCAAAACAACTACCATGCTGCCCATTCTGACGAGAATTATTGT
TGTAATTGTTGTGGTTGTGGTGTCTCT

..((((((((.....(((......)))((((((((((..((.((((((.((((((((....)))))))).((..(((..((.((((..............)))).))..)))..))....))))))))...)))))))))).
))))))))..

****
************************************TTGTTGTAATTGTTGTGGTTG**********

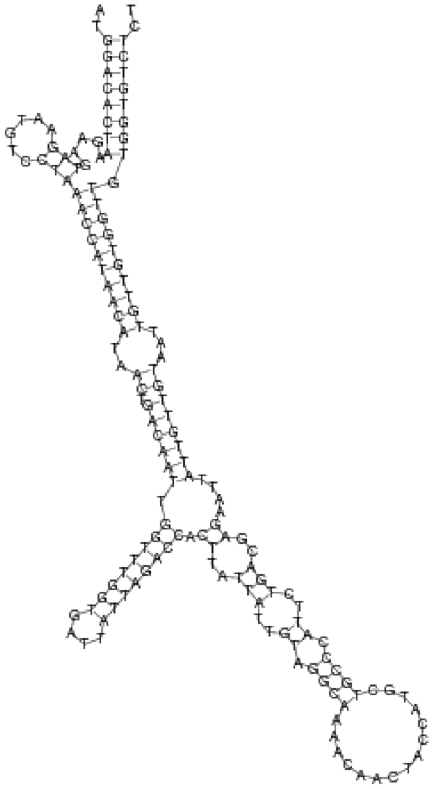

Novel_mir_166 (CK-m0210-5p 20)

TGTTTTTAAGTTCAGAGCAGTCAGACACATCAAGTCTTTTGAGGGATTTCAAACCACGTATGC
TACTGGGAATACTTTCAAGGTTCTTACAATTGTTCATGCTCAATAGAACTAAACCAGCCAAAC
AATGAAATGATGAGGATAGTTCTGCAATAGCAGTCCCATCTAAACGAAGCTCCCTCAAGCAAT
TCATGTTTCCTACTATATCAGGAAACTTATCAAGTTTTGAGCAGCTACTGAAAGTGCAAACT
.((((.((..((((((((.((((((.((.........((((((((((((.......((.((...(((((((....)))..((((((....((((........))))))))))...)))).)).)).........((((.(((..
...((((....)))))))))))).....)))).)))))))))........(((((
**********TTCAGAGCAGTCAGACACATC**
****
****
*

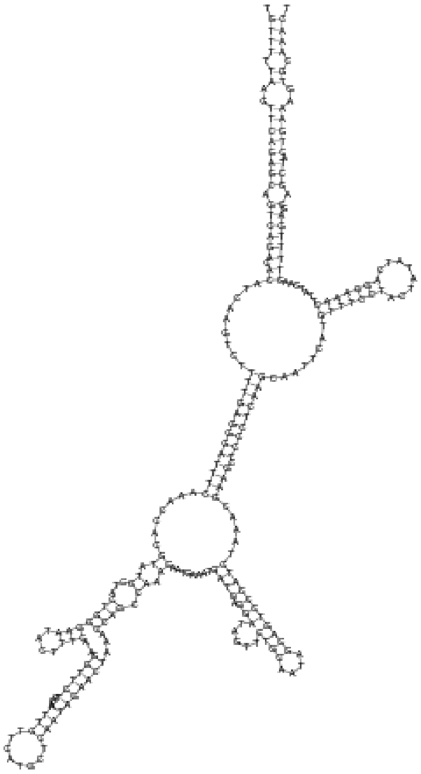

Novel_mir_191 (CK-m0242-3p 27, Sb052-m0230 11,Th053-m0241 25)
GCAGCTTTCACCACCCCATCAACTCCTCTCTTTCTCTCTCTAAAACCCCCTTCTCTCCTTCTTTA
AACCTCCGACTCCGCCCTTTCTTATTACCTTGTACATTGCATCCTGACAATGCTGACCCTGTTT
CGGAAACAGTCCCTCCGATTTCAAATTCCAATAAAACACAAGAGGTGGTGGATGTAGTGGAG
AGCAATGAGAGTGGAAGGCAGGAGGAGGAAGGGCAGGGAGGCAACTTAGTGGAGGAGAAA
GAAGGTGGTGGTGGGGTTTATGATAGTAAT
...(((.(((..(((((((((.(.(((.((((((((.(((((.....((((((.((((((((.....(((.....(((((....(((((((..(((...((((((((((...............((..(((((.........))
)))...))............(((.....)))..))))))))))..))).)))))))))))))))).))))))))))))))....(((....)))..)))))))))))))))).).))))))))...))).)))...
****
****
***AGAAG
GTGGTGGTGGGGTTTA**********

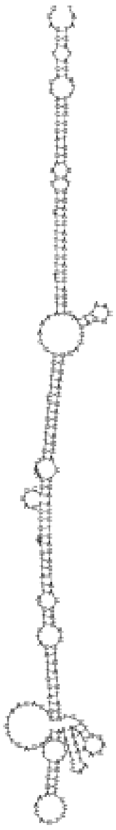

Novel_mir_244 (CK-m0347-5p 18, Sb052-m0332-5p 8)
CTCCGCAGCCAGGGATTCTAGTTGTTTACGGTCATCTACATCATCAAGAACAACAAGAATCTT
TTTACGTTGTAACT
....(((((.(((((((((.(((((((..(((.((....)).)))..))))))).)))))))))....)))))....
**********AGGGATTCTAGTTGTTTACGG**

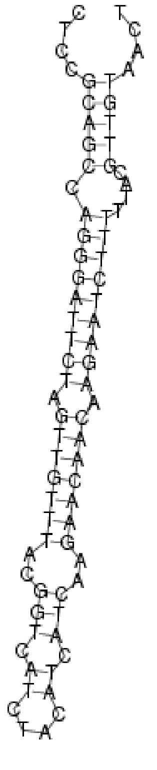

Novel_mir_248 (Sb052-m0010-5p 22)

ATATGAGGGAGAGGATTCTGATGACGGTGGTTTCAACGGCCGTTGATGGCTGATGAGGGAGA
ATCGTCGATTTGAAGCTTCGGGTGGCCGATGAAAATCGCGACCTTTTTCGGCCAGATAGGGA
AGAGAAAACACTGAAACTGGTCGGGTTTTTGCTGAATAGGAGGCTAGTAACCTTTCTATGGTG
GTTTGGAGGCTTCAAACGGCCGGAAATGGGAGATCAGATGAGAGAGAGAGAATCTCCGGTGA
GAGGAGAGAGAGACTCCGAGATTGTGTTACGGTGTGAACGTTAGCATGGTACACCGGTGCAG
CTGAAGGTTGTGAACAGTTGGATCGCGGATGAACTAATCCTTCGGCT

...((((((((((.(((.(((((((((..(((((.((((((....)))))).)))))....)))))).))).))).))))..(((((((((.....((((((((((.(((((((((...((............))....
)))))))))).....((((....((((.((.....((((((..((((.(((((((...))))))).))))...((.(((((((.............).)))))).))))))))....))...)))).........((((((
(..(((....))).)))))))...)))).)))))))))))...))))).))))...........)))))))...

**********GAGGATTCTGATGACGGTGGT**
****
****
****
****

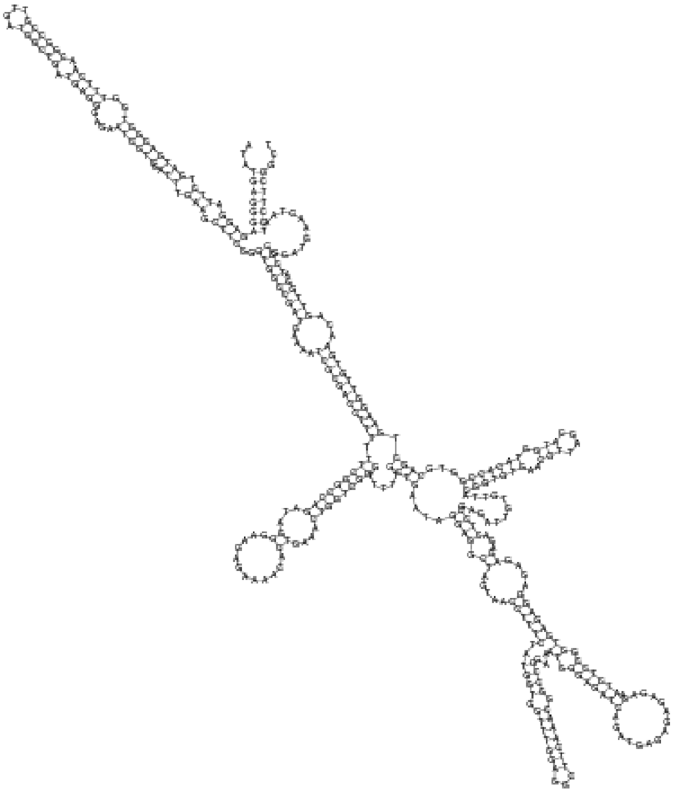

Novel_mir_206 (CK-m0266-5p 184, Sb052-m0251-5p 161)

TATATACCGACGGAATATTTTCGTCGGTAATTCCGTCGGTATATTCCGACGGAAATATTCCGTC
TGTATATACCGACCGATTTGGAGACGGAATTATGTCCGTCGGTAATCATTACCGACGGAATAT
TTCCGTCGGTAATTC

....(((((((((((((.(((((((..(((.((.((((((((((.(.((((((.....)))))).).)))))))))).)).)))..))))))).))).))))))))))...((((((((((((.....))))))))
)))..

**********CGGAATATTTTCGTCGGTAAT**
****

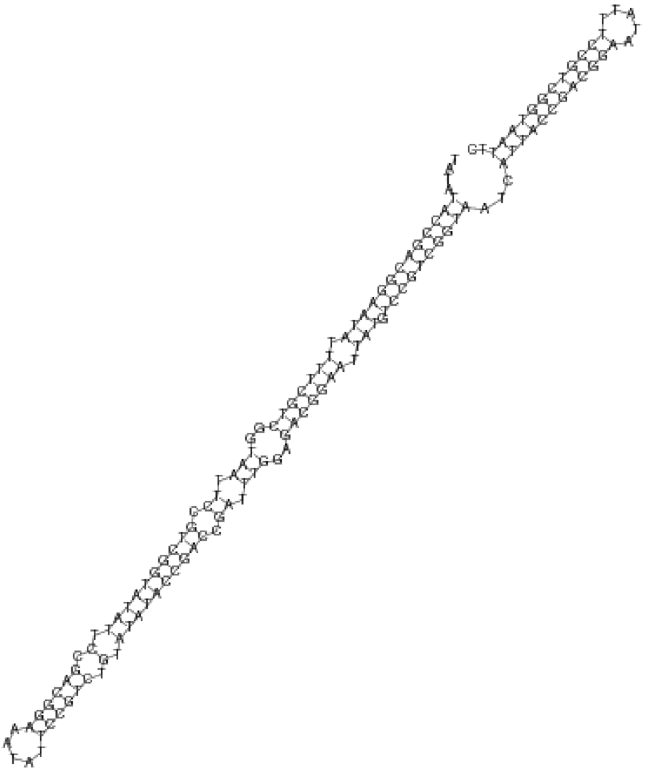

Novel_mir_211

(CK-m0274-3p 27, Sb052-m0264-3p, 16 Th053-m0273-3p 14)

TTTGGTAGCATCAAGTTCATCATCCAATTTGGAAATTTTATGGGGGAAGTGGCAGCTTCCTCA ACTTCTTCAGATTTGAATGAGGTTGAAGCCTATCAA CK-m0274 27

.((((((((.((((.(((((..((.((((((((.........((((((((....))))))))......)))))))).))))))).)))).).)))))))

TTCAGATTTGAATGAGGTTGA********

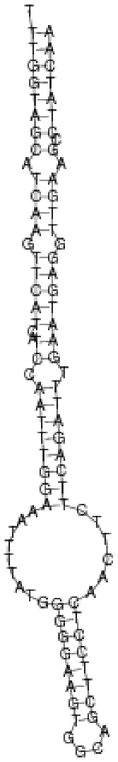

(CK_m0275_3p 27, Th053-m0274-3p 14)
ATGGTGGAAGTTGTTCACAGTTGTAACAGTCTCTTAGCTTCAACTCCACTAGATTTGGTAGCAT
CAAGTTCATCATCCAATTTGGAAATCTTGAACCTCCATATTCATCTATTCTCAACGTCTTCAGA
TTTGAATGAGGTTGAAGCCTATCAA
.(((((((.((((.....(((((...........))))).)))))))))))...((((((((.((((.(((((..((.((((((((.((.((((...................)))).)).)))))))).))))))).)))
).).)))))))
****
************************************TTCAGATTTGAATGAGGTTGA**********

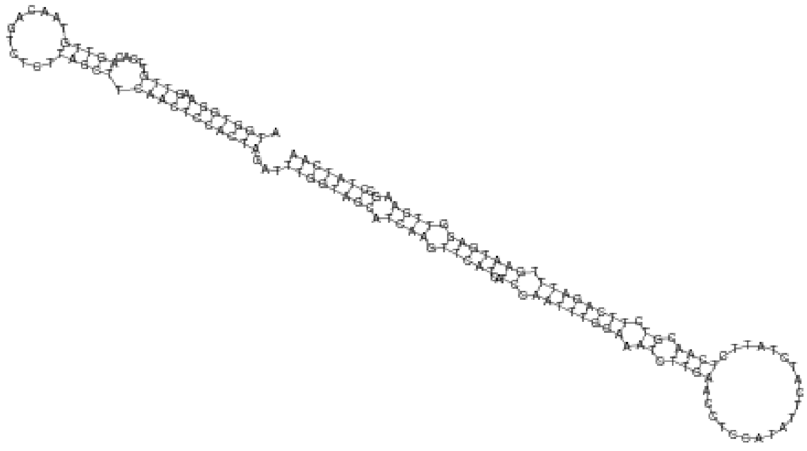

Novel_mir_222 (CK-m0292-5p 11, Sb052-m0277-5p 20)
AGACTTTCACTTGAGGGTGTGTGTTAGAGAATAATATAAAATCATATATCGAAACCTCACCTA
ACAGTTTAAGCTTTTAGGTTAAATTGGTTCTTTGACAAAGTAAACATATTCATCCAAAGCATC
ATACAGAACATACAACCATCACAGTATTCAATCACCTAAGTTATTGCTCTCTTTGTTATGCTGT
CATTTTCTTCTATAATTTATTAACTTAAATATTAAAGGATCTTTATTTTATAGAGAACTTATTTT
GCAATGAACATAAAGGTCTAATTCAATCCAAATTTCTTACAACTTCACCGTACAAGTATCGTT
TA
((((....((((((.((((..(((.(((((...(((((......))))).(((((((.((((((.(((....))).))))))).......((((.(((.((((((((.............(((((.(((.(((..((((....
......)))).((((.((....)).))))...))).))).)))))......((((.((((((..((....(((........)))......))..))))))))))).)))))))))))).))))....))))....)))........)
)))).)))....)))).).)))))...)))).
**********TTGAGGGTGTGTGTTAGAGAA***
****
****
****

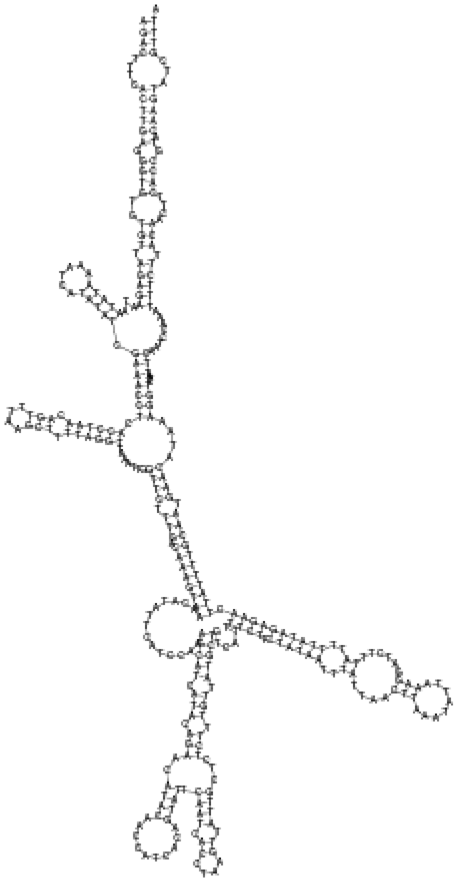

Novel_mir_25

(CK-m0028-5p 75, Sb052-m0023-5p 55, Th053-m0028-5p 18)

AAAATATTGACGGAATATTTTCGTCGGTAAATTACAGTAAATTTTACCGATGAAAATATTCCC
TCGAAATATA

......((((.((((((((((((((((((((...........))))))))))))))))))))).))))......

**********CGGAATATTTTCGTCGGTAA**

GAAATACCGACGGAATATTTTCGTCGGTAAATTTCTGAGGGATTTTACCGACGGAATTACCGT
GGAAAAAAAATTTAAAACAAAGCAAAAAAAAAAAAGATGACGTGTCATTTTTACCGACGGAAT
CACCGATGGAATTCCCAACGGAATATTCCGTCGGTAAAATCCCTCGGTGATTCCGTCGGTAAA
CTGTGAACACTGTTCATCATGTCAATTACAAAGGAAATCACCGACGGAAATTTCCGTCGGTAT
TTTC

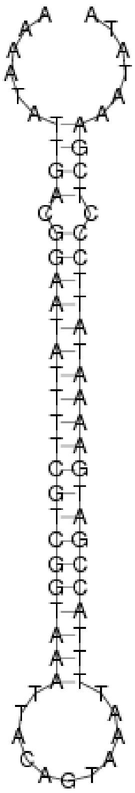

(CK-m0247-5p 75, Sb052-m0235-5p 56, Th053-m0250-5p 18)

(((((((((((((((.((((((((((((..((((((...(...((((((((((((((((((.((...............................(((((....)))))((((((((((((((..(((.(((.....))).

)))...))))))))))))))...)).)))))))))))))))))))..((((((...))))))...........)...)))))).))))))))))))))))))))))))))))).

**********CGGAATATTTTCGTCGGTAA**

****

****

****

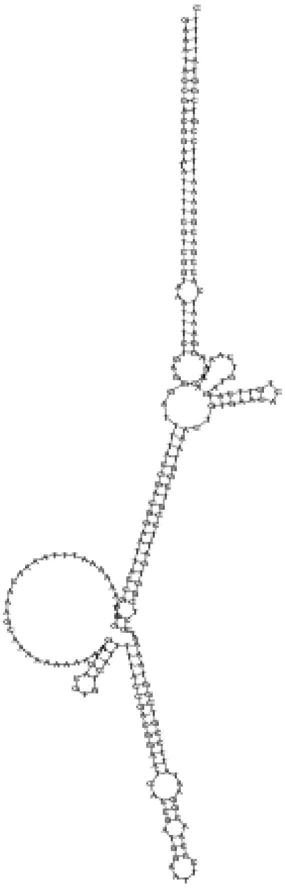

(CK-m0302-5p 75, Sb052-m0286-5p 56, Th053-m0294-5p 18)

GAAATACCGACGGAATATTTTCGTCGGTAAATTTCCGATTCCCTCGGTATATACCGACGGAAT
TACCGTGGGAAAAAAAATTTAAAACAAAGCAAAAAAAAAAAATGACGTGTCATTTTTACCAACG
GAATTACCGACGGAAATACCGACGGAAAATTCCGTCGGTAATTTCG

((((((((((((((((.((((((((((((..((((((.(((((.(((((....((...))...))))).))))))........................((((((((....))))))))....(((.....))).))))))))
))))))))))))))))))))).))))).

**********CGGAATATTTTCGTCGGTAA**
****
******

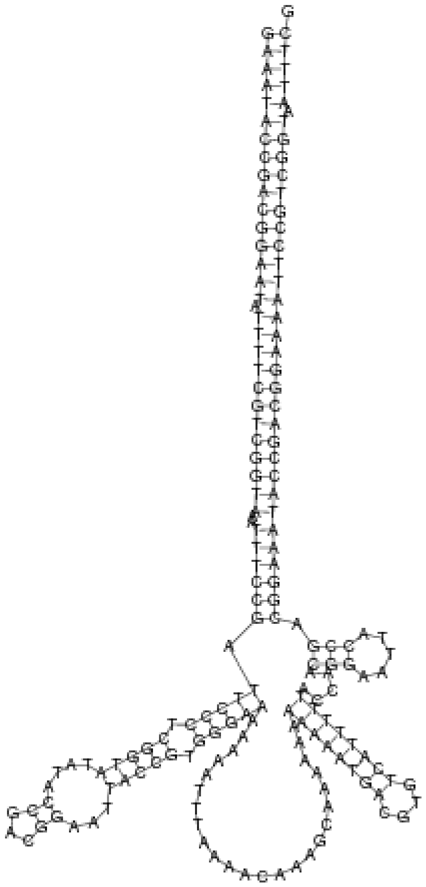

Novel_mir_250 (Sb052-m0015-3p 18, Th053-m0022-3p 21)

ATTTTTTTCCAGTATGTCAAGTGCATCATCATGATGCCATAGTCTTGTACGTTTCCCAAGGTCATCGGGTGATTCTTGACGGACAATTTCTCTCC

...........((.(((((((...((((((.(((((((.....................)).))))))))))).))))))).))...........

TCGGGTGATTCTTGACGGACA*******

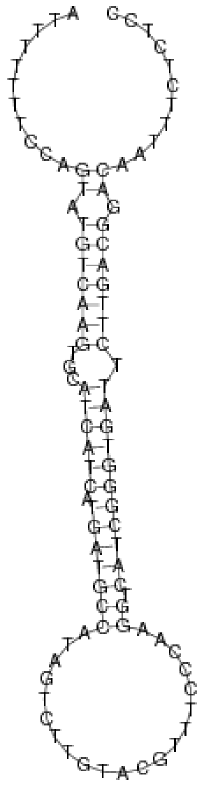

Novel_mir_270 (Sb052-m0070-5p 30)

TCCTCTTCTTTCTTTCTCTCGGTTTTCTTCTCCTCTCTTCTCAAATTTTGCTACTGCCATCTACAG
TAGCCGGACATTTAATTTAAACCAAAGAGGCAGCCAGCTGCCTCCTTAATTAATGTGGTGGTC
CCCCCATCACATGGTGCGGGACCACACAACAAAAGGAATGGCGGGTTTCATCTTTCGGAAGA
AGGGAAAAGCCTCCTCAATTTCGTATGCTCCTATCCTTGTATTTCGTCTAAAGAAAAGATATTA
CTCTACTATGTCTATCAAAGAATAGTCTAATATTAATTCAGGACATTTTTAATATTTCAGACAT
GGAAGCTGTGGTCTGAAGGTAAAGGACTGGAG

(((..(((((((((((.((((((((((........((((..........(((((((.......))))))).((((((..........((...(((((((....)))))))........(((.(((((((((((((...)))).
).))))))))...)))...))...((.((((((..((((((....))))))..))))))))........................((((((..((....))..))))))........))))))....))))...(((((((((((
(............))))))))..)))))..)))))))).))...))))).))))))..))).

*********TCTTTCTCTCGGTTTTCTTC***
****
****
****
****

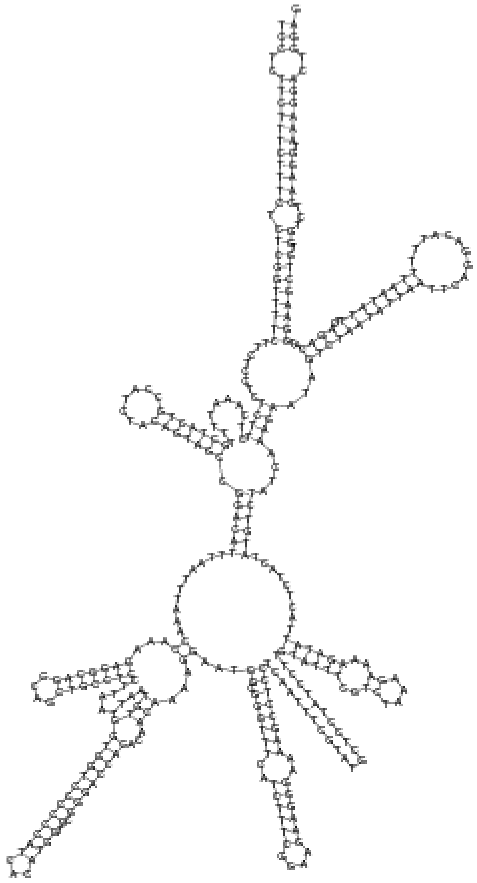

Novel_mir_290 (Sb052-m0125-5p 21)
AAGACGGCGAGATGATTTGGAAGCCGGAGAAAGTCGTTCTACTGGCTTTGATCGCCTCCATCG
GCCGCCTC
.((.((((((((.(((((..((((((((((((.....)))).)))))))))))))).))).....)))).)).
**********GATGATTTGGAAGCCGGAGAA***

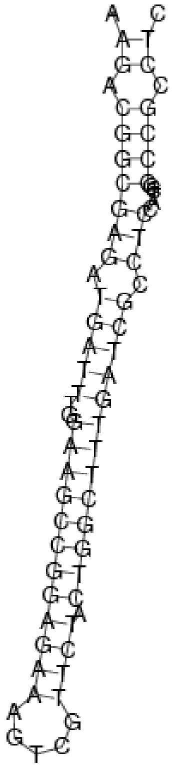

Novel_mir_357 (Sb052-m0316 32)

AGGAATTTACCCCTTCTTCATCTGATCTTGTTCCTATTATCTCCTCCATTTTCTTTTATTCTTCAA
CTCTAATCTCTTCCAAGTTTACGAGGTTCGGCAGCAACACAAGAGGGAGCAATTTCTTCAAAC
TCTTACAACCAGAGCATTCAAACTCTTTAACACTAGAACATATACCATGATAAGATGGAGATG
ATGGTGGAGCAGAGCAGAACCAAGAAAATGAAACCA

.((..((((....(((((..((((.(((.(((((.((((((..(((((((((...((((.......((((((.((......)).))).)))((((...........(((((((((......))))...)))))......((((.
.......)))).........)))).......)))).)))))))))..))))))))))))).))))...))))).)))).)).

***************CTTCATCTGATCTTGTTCCTATT***
****
*** Sb052-m0316-5p 1
****
****
************************TGGAGCAGAGCAGAACCAAGAA********** Sb052-m0316-3p 31

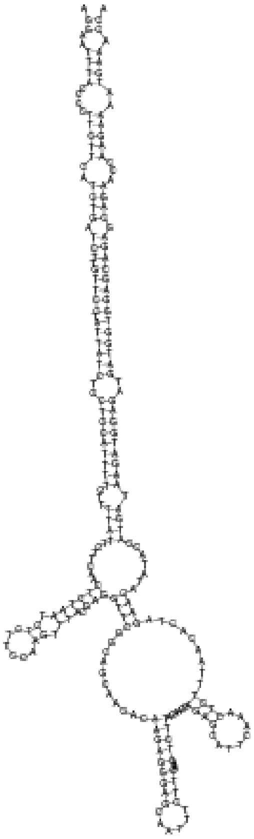

Novel_mir_368 (Th053-m0023 4282)

CTCATGAAATCCAAGTGGACTTCCCTTACAATCTACTCTTTCAGTTTCACAGAAGATCCAGATT
AGATTTTAAGGCAAACAAATTGATAGAGCAGATTGTAAGGGAAGCCCACATGGATTTCAACC
C

....(((((((((.((((.(((((((((((((((.((((.(((((((..(..((((((.......))))))..)......))))))).)))).))))))))))))))).)))).)))))))))....

************AAGTGGACTTCCCTTACAATC***
**Th053-m0023-5p 154

****
**********TTGTAAGGGAAGCCCACATGG********** Th053-m0023-3p 3959

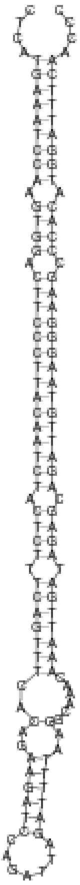

Novel_mir_38 (CK-m0043-3p 32, Sb052-m0039-3p 25, Th053-m0047-3p 9)

ACACTTACAAGTATTGAGCATATTCTAGATGATTTCTTAAACGTACTAACGCAAATATCTTCCA
AGAAATATTTAATTAGAATTCATCTTGTTTTCTCAGAAGATTTCAAGCTTGACACTATTGCCTT
ATTTGACACAGGTGCAGATTTAAACTGTATCAAAGAAGGCGTGGTTCCAAAATTTTTTTTACA
AAATACATCTGAAAAACTCTCTGCTGCTAATAAT

.........((((..(((....(((.(((((((((..((((.........((....((((((..(((((......................)))))..)))))).....)).((..(((((.(((((.(((((.((((..........
...)))).))))).))))))))))..))........))))..)))).))))))))...)))..)))).........

****
****
********************ATACATCTGAAAAACTCTCTGC**********

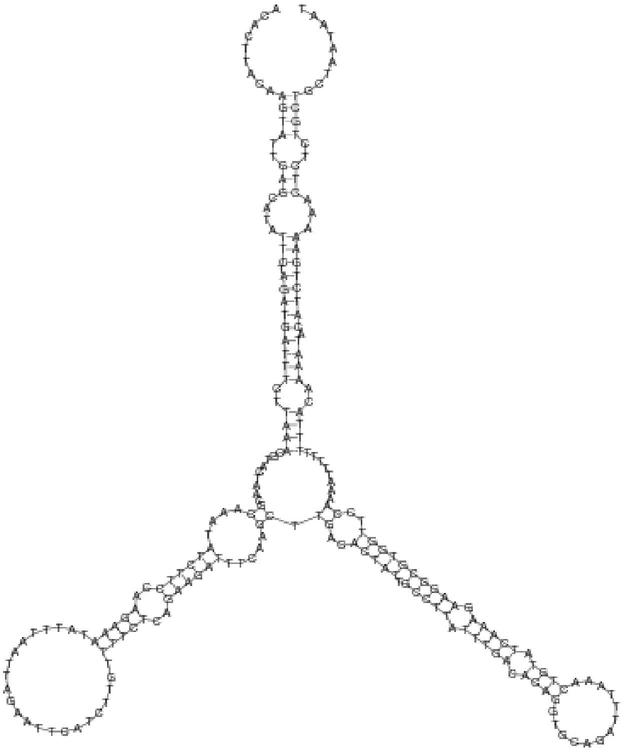

Novel_mir_387 (Th053-m0092-5p 22)

GAGAGTTTGGTGTTAAGGTTTGATGGGAAAGATTTATTTGTAGTGTAAGCAGATGAAGTCTTA
CATAAATGGTATTGGTATTCAACAATTTACTGGAAGTAGTTCTTTCCCATCAAACCTCAACATT
ACGCTCTA

.(((((.(((((((.(((((((((((((((((.((((((.((((((((((.......).))))).........((((........))))..)))).)))))).))))))))))))))))).))))))).))))).

**********TGTTAAGGTTTGATGGGAAAG***
****

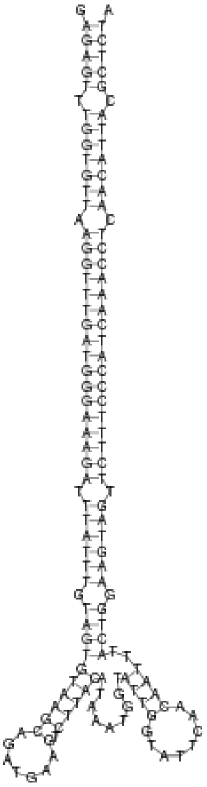

Novel_mir_403 (Th053-m0153-3p 21)

AAAGATTTACATGGTTATTAATTTTAGAATTCATGAAATTAATCAAGATATATGTGAACTGGT
ACGAATAATTATGTTAATAAAAAAAAAAAAGTTAAGGTATAGAGATATTTCTTAGAATTATCAA
ACCTAGGACAAATTGTCCTTTAATCTTGACATTGATGAAGGGAGTGTCTAGAATTGCAGTGAA
AGTTGTGGTTTAAAGTATTTTTTTGTTTGAAAATAATATTTTTTTATTTTTAAAAATTATTTTTG
AAGAGGGTGTTTGAGAGTGTAGTAGCGGTTGTGATTTAAA

...((.((((((.(((((((.(((((((((((.(((......)))............((..((.((((((((.((..((((((((((....((((((((.....((((.(((...))).))))......(((((.....)))
))...)))))))).(..(..(((((((((..(.((((..(((......)))..)))).)..)))))))))..)..)........))))))))))..))..)))))))).)).)).)))).)))))))...))))))).).)
)))).))...

****
****
****
TGTTTGAGAGTGTAGTAGCGGTT*******

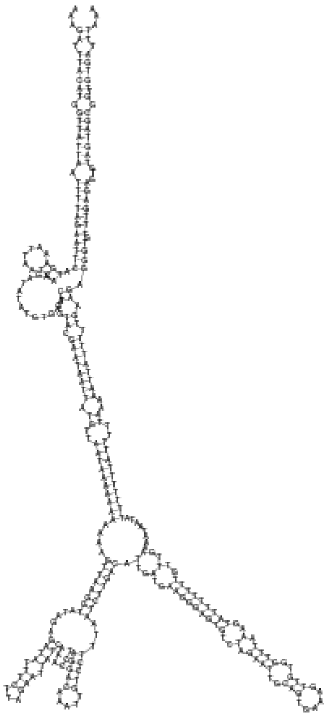

Novel_mir_93

(CK-m0111-3p 82)

ATTTGGGTCTGATTGAAGGATTTAATTAGGTGCAAGGACTTAATTATACTTTAAACATGTCTA
ATTAATTTTCTTAGGGGTTTAATTGGTGAAAACTTAAGTTTGGGAGCCCATTTTGGACTTAATT
GAGAAGATTAGAATTTTAG

..(((((..((.((((((....(((((((((......)))))))))..)))))).))..))))).((((((((((((.(((((((..(((..((((....))))....)))....)))))))...))))))))))))...
......

****
***************************TTTGGACTTAATTGAGAAGATT**********

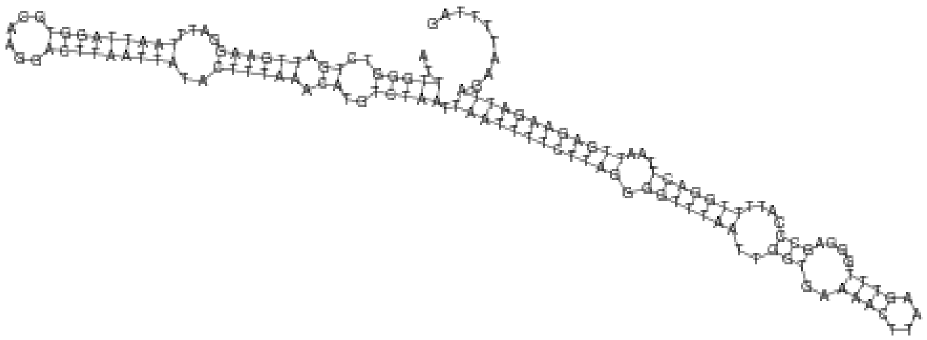

(CK-m0314-3p 151 Th053-m0307-3p 105)

TTAATAAGCCAATTTGATTTAATTATGGGCCAAATTAAAGATTAATTATGTTTAAAAATTAATT
TGGATTCAATTAAAGGATTTAATTAGGTGTAAAGACTACATTATACTTTAAATGGGTCAAATT
AATTTTATTAGGACTTAATTGGTGATTAAATGGGTCAAATTAATTTTATTAGGACTTAATTGGT
GAAAAATTAAGTTTAGGAGCTCACTTTGGACTTAATTGAGAAGATTGGAATTTTAG
.(((....(((((((..((((((((.((.(((((...((((((((((.......((((((((((((.(((((.((((((....(((((((.........))).))))..)))))).))))))))))))))))).....((
((((.(((.....))).)))))).))))))))))....((((((((((.......))))))))))..........))))).)))))))))).)))))))....))).
****
****
TTTGGACTTAATTGAGAAGATT*******

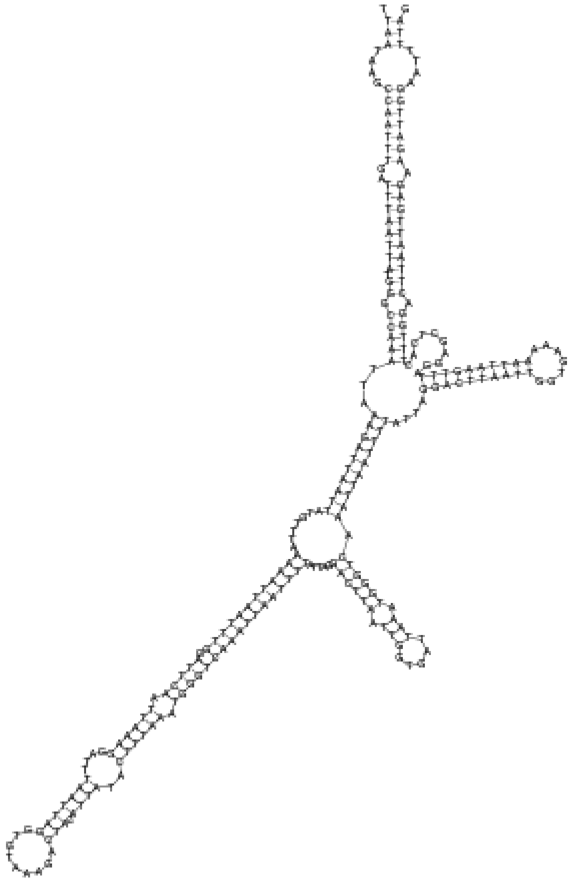

Supplement: Additional file 3: Figure S1. — Precursor sequences and the predicted secondary structures of the 20 novel miRNAs from P. szechuanica (“(”represent base matches, “.” represent base mismatches). (PDF 387 kb) [file 12864_2015_1891_MOESM3_ESM.pdf]
